# Reevaluating Mask Effectiveness: Insights From Evidence-Based Medicine and Clinical Trials

**DOI:** 10.7759/cureus.75455

**Published:** 2024-12-10

**Authors:** Beny Spira

**Affiliations:** 1 Microbiology, Instituto de Ciências Biomédicas, Universidade de São Paulo, São Paulo, BRA

**Keywords:** covid-19, evidence-based medicine, masks, observational studies, randomized clinical trials

## Abstract

During the COVID-19 pandemic, masks were widely promoted and mandated as a key measure to help reduce the transmission of SARS-CoV-2. These policies were primarily informed by laboratory evidence demonstrating the effectiveness of particle filtration, alongside observational studies. While several meta-analyses have indicated that masks may contribute to reducing viral transmission, many of these analyses rely heavily on observational data. There also appears to be a trend where the inclusion of more randomized controlled trials in a meta-analysis is associated with a lower estimate of mask effectiveness. It is important to recognize that success in laboratory settings does not always directly translate to the same outcomes in clinical trials or real-world conditions. This phenomenon is often seen in drug development, where therapies with promising mechanistic evidence may not always perform as expected in trials. In this regard, masks share similarities with other interventions that, while theoretically sound, require further testing in varied contexts to fully assess their real-world impact.

## Introduction and background

In spite of significant progress in biomedical sciences, many health concerns remain unresolved. To ensure the safety and efficacy of new drugs and therapies, researchers have developed efficient, though not bulletproof, methods. Randomized controlled trials (RCTs) are the most rigorous and reliable way to determine the efficacy of new treatments or experimental drugs and are therefore considered the gold standard of clinical trials [1,2]. Drug licensing authorities rarely approve a potential therapeutic molecule without a properly conducted RCT [3,4]. Currently, the FDA (US Food and Drug Administration) and EMA (European Medicines Agency) consider observational studies reliable sources of evidence only when their effects are sufficiently large or dramatic [5].

## Review

RCTs on masks

The effectiveness of face masks in curbing viral transmission has been tested in several RCTs before and after the onset of the COVID-19 pandemic. Two large RCTs on COVID-19, the DANMASK [6] and the Bangladeshi [7] trials, have been conducted and published. The DANMASK RCT carried out between April and May 2020 in Denmark involved the largest set of participants (slightly less than 5000) in a mask RCT up to that time. Results of the trial showed a non-significant reduction in the odds ratio of the masked group compared to the non-masked control group (OR = 0.82 [0.54-1.23]; p = 0.33). The authors of the study concluded that “the recommendation to wear surgical masks to supplement other public health measures did not reduce the SARS-CoV-2 infection rate among wearers by more than 50% in a community with modest infection rates, some degree of social distancing, and uncommon general mask use.” The reference to a 50% reduction was because the trial was powered to find at least this degree of risk reduction. Even though the RCT involved a large number of participants and was well-conducted as possible by a pro-mask advocate principal author [8], the mainstream medical-scientific community did not spare efforts to diminish the study's impact on the ineffectiveness of masks in reducing COVID-19 [9-12].

Later on, a cluster RCT on the effect of mask wearing on COVID-19 transmission was carried out in 600 villages in a Bangladesh rural zone. This trial contained the largest number of participants (300,000) ever used in mask RCTs. Tests with both cloth and surgical masks showed that the former had no effect on viral transmission, while surgical masks reduced SARS-CoV-2 seroprevalence by 9.5% and COVID-like symptoms by 11.6% (both values significant at the 5% level). In total, there were 1086 cases of symptomatic seropositive infections in the control arm and 1106 in the treatment arm. This limited reduction was hailed by part of the medical-scientific community as a definitive proof of masks’ effectiveness [13-15]. However, even this small effect is disputed, since a reanalysis of this RCT found that unblinded staff may have contributed to significant bias in the way the treatment and control groups were approached [16]. A surprising finding in this study was that masking effectiveness differed considerably depending on the age of the wearer. While the 50-59 and ≥ 60 year-old groups displayed significant reductions in COVID-19 seroprevalence (0.772 [0.595-0.949] and 0.647 [0.448-0.845], respectively), the younger age groups, (< 40 and 40-49 years old), did not show any significant reduction in seroprevalence (0.967 [0.834-1.100] and 1.009 [0.817-1.200], respectively). The authors of the paper provided some hypotheses to explain this discrepancy, but none have been hitherto tested. More recently, a randomized trial comparing the effect of medical masks and N95 respirators to prevent COVID-19 in 29 healthcare facilities in Canada, Israel, Pakistan, and Egypt has been published [17]. No significant difference between the N95 and medical mask arms in the intention-to-treat analysis was observed. RT-PCR confirmed that COVID-19 occurred in 52 of 497 (10.46%) participants in the medical mask group versus 47 of 507 (9.27%) in the N95 respirator group. The pooled hazard ratio was 1.14 [0.77-1.69]. A no-mask group was not included in this trial. This was the largest RCT on N95 respirators ever performed.

The Cochrane review on mask RCTs

Most randomized trials conducted before the COVID-19 pandemic have reported null to low effect of masks and no difference between masks and respirators. The best RCTs of this kind were systematically reviewed by Cochrane researchers. The fifth and most recent review was published in January 2023 [18]. Cochrane is an international network whose mission is to analyze and summarize the best evidence from biomedical research providing authoritative and reliable information, unconstrained by commercial and financial interests [19]. Cochrane reviews are internationally recognized as the benchmark for high-quality information about the effectiveness of health care. Over 7,500 reviews have hitherto been published, they are freely available from the Cochrane library [20]. In the 2023 review on masks and other interventions, the authors analyzed 13 RCTs on medical or surgical masks, of which 11 were in the community and two were among healthcare workers. In addition, five studies on N95/P2 respirators (four in hospital or outpatient settings and one in the community) were also evaluated. All of these trials studied the spread of influenza or influenza-like illnesses (ILI)/COVID-19-like illnesses. Medical or surgical masks were compared to a no-mask group and respirators were compared to medical or surgical masks. The most reliable outcomes were from studies that used laboratory-confirmed viral illness, which provided a “moderate certainty of evidence”. Overall, the evaluated studies showed no significant risk reduction by medical/surgical masks or P2/N95 wearers. The total number of participants in the RCTs on medical/surgical masks versus no mask was 290,000, with approximately half this number in each arm. The pooled risk ratio (RR) for laboratory-confirmed influenza was 1.01 [0.72-1.42], while the pooled RR for influenza-like illness was 0.95 [0.84-1.09]. A pooled RR of 1.10 [0.90-1.34] was observed when N95 respirators were compared with medical/surgical masks in laboratory-confirmed influenza, a result that surprisingly favors surgical/medical masks, but without statistical significance. Conversely, the two other illness outcomes, clinical respiratory illness and influenza-like illness, which provided less certainty of evidence, also showed statistically non-significant results, but with pooled risk ratios favoring N95 masks (0.70 [0.45-1.10] and 0.82 [0.66-1.03], respectively). Two RCTs showed statistically significant results in favor of masks [7,21]. The Bangladeshi study did not provide exact p values but stated that the overall effect of surgical masks (11% RR) was statistically significant at the 5% level. Another RCT [21] demonstrated a significant level of confidence (p = 0.0238) in one of the interventions analyzed, revealing that medical masks reduced clinical respiratory illness, though not ILI or laboratory-confirmed viral illness.

In general, there is an inverse correlation between the mask study quality (or quality of evidence) and mask effectiveness, i.e., the better the study or the quality of evidence, the lower the effectiveness of masks. This becomes evident when more rigorous and less rigorous studies are compared (e.g., randomized vs. observational studies) or when comparing different endpoints: clinical respiratory and influenza-like illnesses (less reliable endpoint) vs. laboratory-confirmed influenza (more reliable endpoint).

The Cochrane review on mask RCTs [18] concluded that “The pooled results of RCTs did not show a clear reduction in respiratory viral infection with the use of medical/surgical masks. There were no clear differences between the use of medical/surgical masks compared with N95/P2 respirators in healthcare workers when used in routine care to reduce respiratory viral infection”. It is worth noting that there were no qualitative differences between the current and previous Cochrane review published in late 2020 [22] despite the inclusion of three recent trials [6,7,23], two of them on SARS-CoV-2 transmission [18]. If anything, the effect size of masks decreased with the addition of these trials. Additionally, all but one N95 trial, including the most recent international multicenter RCT [17], was conducted with healthcare workers who had been trained on how to fit and wear respirators. As these trials have not demonstrated a reduction in viral transmission when compared to surgical/medical masks, it makes one wonder if the recommendations to wear respirators and the calls for their distribution to the general public during the COVID-19 pandemic [24-26], who are not trained to use these types of masks, would have been of any use.

Meta-analyses that support the use of masks

Since the early beginning of the COVID-19 pandemic, several systematic reviews and meta-analyses evaluated the data from mask trials [27-36], and most of them concluded that mask wearing was associated with a significant reduction in viral transmission. For instance, a meta-analysis published in December 2020 (at the same time as the Cochrane review), examined 12 randomized trials and 21 observational studies on the effectiveness of masks in preventing the transmission of respiratory viruses [35]. According to this review, face masks reduced the risk of primary respiratory infections by 6 to 15%. The authors justified the inclusion of observational studies because "the conclusions of RCTs and observational studies differ widely and both are prone to significant bias". However, since randomized trials are designed to minimize bias and confounders, their inherent bias is considerably lower than that of observational studies [2]. Additionally, the risk reduction calculated in this meta-analysis (6-15%) for mask wearers was unimpressive. An exception to the pro-masking wave of meta-analyses that reviewed observational studies is Wang et al. [36] who, by analyzing five randomized and 10 observational studies, estimated a non-significant pooled odds ratio of 0.96 [0.8-1.15] for surgical mask effectiveness against acute respiratory illness among individuals in non-healthcare settings, concluding that “surgical mask wearing among individuals in the community is not significantly associated with a reduction in acute respiratory illnesses”.

**Figure 1:**
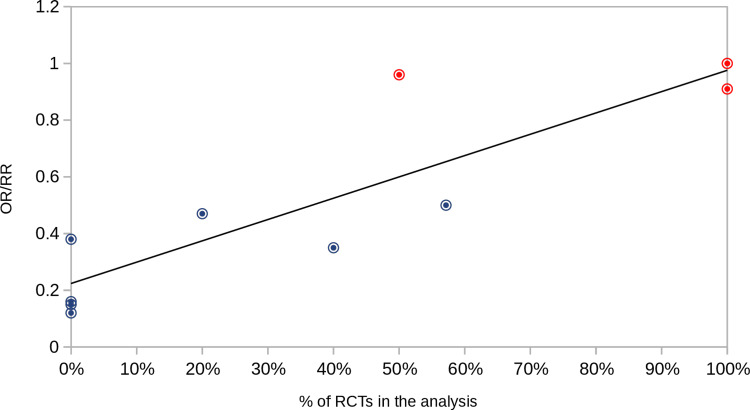
Correlation between the proportion of RCTs in 10 different mask meta-analyses and the respective pooled effect sizes. The effect sizes (odds ratio (OR) or risk reduction ratio (RR)) of 10 meta-analyses on masks and respiratory viral illnesses, all published since 2020 [18,24-34,36], were correlated with the proportion of RCTs or cRCTs included in each analysis. Given the overall low incidence of cases in the meta-analyses, RRs were very similar to ORs and were therefore used interchangeably [40].  An individual meta-analysis is represented by a bullseye symbol. The red symbols indicate no statistically significant reduction in the primary outcome (lab-confirmed illness or influenza-like illness). RCT: Randomized controlled trial; cRCT: cluster randomized controlled trial

Meta-analyses that concluded that masks do have a substantial impact on viral transmission relied only or almost exclusively on observational studies. This is exactly what the very influential paper by Chu et al. [27] has done. This review pooled the outcomes of 29 observational trials (26 in healthcare settings and three in the community) and not a single RCT, reaching a significant adjusted odds ratio of 0.15 [0.07-0.34]. Subsequently, other meta-analyses were published focusing primarily or exclusively on observational studies [25,30-34]. All of them concluded that masks were effective in preventing respiratory infections. Figure 1 shows a correlation plot between the proportion of RCTs in ten mask meta-analyses and their respective pooled risk reduction ratios (RR). The key details of the meta-analyses used to create Figure 1 are presented in Table 1. It can be observed that the lower the proportion of RCTs in a meta-analysis, the greater the effect of masks (lower RR). As expected, there is a strong association between these two variables (Pearson correlation coefficient = 0.88). It should be noted that two systematic reviews [18,26] that included only RCTs in their analysis and the meta-analysis conducted by Wang et al. [36] that contained both RCTs and observational studies found no statistically significant effects. Two systematic reviews containing mostly RCTs were not included in this analysis: studies by Chaabna et al. [37] and Li et al. [38]. The former included 11 studies: nine RCTs, one observational cohort study, and one case study, but two of the RCTs they analyzed did not have a no-mask group, the comparative analysis was between a mask group and a mask + hand hygiene group. The other review [38] analyzed eight RCTs and reported a somewhat positive effect of masks on reducing respiratory infection (OR = 0.84 [0.71-0.99]). However, this conclusion was based on a single RCT that showed a statistically significant outcome [39]. Unfortunately, this study was not properly randomized [18] and was therefore not included in the present analysis. The meta-analysis by Brainard et al. [35] was not included as well because no pooled effect size estimates have been provided (the authors computed ORs for each category: RCT, cohort, case-control, and cross-sectional studies and also separated ORs primary infections and secondary attacks). 

**Table 1:** Description of the meta-analyses The table includes description of the meta-analyses analyzed in Figure 1. RCT: Randomized controlled trial

Meta-analysis	No. of RCTs	No. of Observational Studies	% RCTs	OR or RR [95% CI]		Reference
1	0	29	0%	0.18 [0.08-0.38]		[24]
2	9	0	100%	0.91 [0.66-1.26]		[18]
3	8	14	57%	0.5 [0.37-0.68]		[33]
4	0	6	0%	0.38 [0.21-0.69]		[30]
5	6	15	40%	0.35 [0.24-0.51]		[25]
6	7	0	100%	1 [0.98 – 1.02]		[26]
7	0	7	0%	0.16 [0.04-0.58]		[34]
8	0	4	0%	0.12 [0.06 - 0.27]		[32]
9	1	5	20%	0.47 [0.29 - 0.75]		[31]
10	5	10	50%	0.96 [0.8-1.15]		[36]

Masking as an “alternative therapy”

As a general rule, the better the quality of the study (e.g., observational vs. randomized trials), the lower the reported efficacy of masks. This rule also applies to clinical trials of "alternative therapies". For instance, Shang et al. [41] and Mathie et al. [42] systematically reviewed homeopathy, a widely used and controversial alternative therapy reaching in some cases small, but statistically significant clinical differences. Here too, there is an inverse correlation between the study quality and the effectiveness of the intervention. Clinical trials that reported positive results for homeopathy also displayed low impact and wide confidence intervals.

A similar pattern emerges when mask trials are examined, with observational studies showing better outcomes for masks than higher quality RCTs, as well as when comparing laboratory-confirmed viral infection with less well-defined clinical illnesses. It should be stressed that even when supported by randomized trials with statistical significance, small effects and low-powered studies in conventional or alternative therapies are dangerously susceptible to biases. 

Masking priors

From a Bayesian perspective, masks should not be regarded as a shaky alternative therapy. After all, intuition, common sense, and most importantly, laboratory experiments provide masks with a relatively high prior probability. It is undeniable that masks are capable of filtering particulates, even if the actual filtration effectiveness of different mask types varies widely across studies [43]. Surgical or cloth masks, which were used by the vast majority of people during the COVID-19 pandemic, provide only 10-12% filtration efficiency. Respirators (P2/N95) are more efficient, but none of them achieve more than 60% filtration, even under optimized laboratory conditions [44]. In addition, a small fractional leak area of 2% can severely reduce mask filtration efficiency by 75%, especially for particles smaller than 5 mm in diameter [45]. Even before 2020, several credible RCTs demonstrated that masks do not perform as well in practice as in the laboratory [21]. If we switch the priors from “in-vitro”/laboratory mechanistic evidence to the gold standard of clinical trials, i.e., systematic reviews and meta-analyses of randomized trials, the prior probability of masks is strongly reduced. In other words, masks are plausible interventions based on laboratory experiments, but their continued failure in randomized clinical trials in reducing viral infection in both healthcare settings and in the community undermines their usefulness, making them similarly ineffective as a questionable alternative therapy. In this regard, the main difference between masks and classical alternative therapies is that the former has been widely supported by the mainstream medical and academic establishment and that alternative therapies have never been mandated by law.

Mask in the lab: filtering efficacy as a secondary outcome

The ability of masks and respirators to filter viral particles under controlled conditions has been examined in a great number of in vitro/laboratory studies. Many of these studies were published during the COVID-19 pandemic after masks were mandated worldwide (see [44-48] for a few examples). These studies also provided material such as images and catchphrases that were widely used to promote masking for the general public. Remarkably, the enthusiasm for the apparent efficacy of mask filtration led some authors to argue that RCTs, the gold standard of clinical trials, should be disregarded in favor of mechanistic effects alone [49] in what was called EBM+ (evidence-based medicine+) where the ‘+’ refers to the addition of non-clinical evidence such as in vitro experimentation (mechanistic evidence) [50]. Their argument goes as follows: “the complex nature of the pandemic, characterized by multiple variables interacting dynamically with a high degree of uncertainty, urgency, and threat to public health, requires the use of mechanistic evidence once randomized trials are difficult or impossible to conduct” [50]. However, there is no evidence to support the assertion that randomized trials are difficult or impossible to carry out. Mask RCTs can be conducted without any hindrance from an operational perspective, as they have been conducted in the past, even during epidemics. Since prior randomized trials have consistently shown that masks do not significantly reduce viral transmission, conducting new RCTs would not pose ethical concerns, as the control group (no masks) would not be exposed to heightened risk. Additionally, the preoccupation with the severity and uncertainty of the pandemic should reinforce, rather than undermine, the need for good and reliable data. This is not the time to disregard the best tool available to the biomedical sciences, the RCT, to distinguish between working therapies and bogus or ineffective ones. The conflict between low-quality data, supported by observation and common sense, and high-quality data permeates the medical literature as shown by Fanaroff et al. [2] who describe 31 heart-related conditions in which common sense or observational evidence predicted positive outcomes, only to be contradicted by robust clinical trials.

Whether a specific therapy or intervention should be implemented cannot be determined solely by mechanistic evidence. The best that laboratory results can do is to provide the theoretical foundation for a successful clinical trial. Furthermore, the biomedical sciences are currently facing a reproducibility crisis, particularly in preclinical research, where numerous studies published by high-impact journals have later proved irreproducible [51,52]. In addition, the fact that a particular drug or therapy is successful in the laboratory does not necessarily indicate that it will be successful in the clinical trial. Many promising drugs with state-of-the-art mechanistic explanations have been withdrawn after failing randomized trials. In reality, less than 14% of pre-clinical potential drugs make it to the market [53], with different therapeutic groups having varying success rates. Oncology-related drugs, which account for the majority of clinical trials, have a success rate (percentage of drugs that are eventually approved by the regulatory authority) of 3.4%. In contrast, biologics such as vaccines are approved at a rate of 33.4% [53].

Some particular therapeutic fields are plagued by an overabundance of failures. Until 2008, there were over 200 failed Alzheimer’s drug candidates [54] and their number keeps increasing. The same is true for other therapeutic areas such as obesity [55] and most other medical fields [53]. All of these potential drugs passed pre-clinical trials with flying colors but failed at one or other stage of the clinical trials. Similarly, mask testing under laboratory conditions may have shown filtration efficacy (although the degree of efficacy is unclear), but clinical trials (RCTs and cRCTs) have repeatedly demonstrated that they are not effective in reducing viral transmission in the community and among healthcare workers. Real-world situations are often considerably more complex than mechanistic experiments conducted under controlled conditions in laboratories, especially since we do not fully understand how respiratory virus transmission works.

## Conclusions

Throughout the COVID-19 pandemic, masks have been promoted as an important tool to reduce or even halt the spread of SARS-CoV-2 in the population. Many governments mandated the use of masks in public by law. However, even before the pandemic, the best evidence available from randomized trials was already showing that masks are likely ineffective in curbing respiratory viral transmission. Additional high-quality data produced throughout the pandemic reinforced that conclusion. While intuition and laboratory experiments suggest that masks are plausible interventions, their continued failure in randomized clinical trials undermines their practical effectiveness.
